# Number of natural teeth and incident cognitive impairment in older Chinese adults: A longitudinal CLHLS cohort study

**DOI:** 10.1097/MD.0000000000051119

**Published:** 2026-10-16

**Authors:** Qihang Chen, Xiaobao Dang, Jingke Liu, Zhen Zhu

**Affiliations:** a Zhenjiang Stomatological Hospital, Zhenjiang, Jiangsu, People’s Republic of China.

**Keywords:** CLHLS, dietary diversity, incident cognitive impairment, longitudinal cohort, mediation analysis, natural teeth

## Abstract

This study aimed to examine the longitudinal association between baseline number of natural teeth and incident cognitive impairment among older Chinese adults in the Chinese Longitudinal Healthy Longevity Survey and explore whether dietary diversity partially mediated this association. We included 4364 participants from the 2008 Chinese Longitudinal Healthy Longevity Survey who were free of cognitive impairment at baseline and had at least 1 follow-up cognitive assessment through 2018. Incident cognitive impairment was defined as a Mini-Mental State Examination Score <24 at any follow-up assessment. Cox proportional hazards models were used to estimate hazard ratios (HRs) and 95% confidence intervals (CIs) for the association between baseline number of natural teeth and incident cognitive impairment, with tooth count modeled both continuously per interquartile range (20 teeth) increase and categorically by quartiles. Restricted cubic spline analyses were performed to examine potential nonlinearity, and subgroup analyses with multiplicative interaction tests were conducted to assess potential effect modification. Over a median follow-up of 6 years (24,585 person-years), 1835 participants (42.0%) developed incident cognitive impairment. In the fully adjusted model, each interquartile range increase in natural teeth was associated with a lower risk of incident cognitive impairment (HR = 0.86; 95% CI: 0.78–0.95; *P* = .002); participants in the highest tooth-count quartile had a lower risk than those in the lowest quartile (HR = 0.81; 95% CI: 0.70–0.93; *P* = .004). Restricted cubic spline analysis supported an overall association (*P* = .013) without evidence of nonlinearity (*P* = .467). Age modified the association (*P* for interaction = .043); the association was evident among participants aged ≥70 years (HR = 0.73; 95% CI: 0.66–0.82) but not among those aged <70 years (HR = 0.95; 95% CI: 0.75–1.19). Exploratory mediation analysis suggested a small indirect effect through Dietary Diversity Score (indirect effect = 0.0012; 95% bootstrap CI: 0.0001–0.0024), accounting for approximately 9.9% of the total association. A higher baseline number of natural teeth was associated with a lower risk of incident cognitive impairment among older Chinese adults. Exploratory mediation analysis suggested that dietary diversity may account for a small part of the association between natural tooth count and mean follow-up Mini-Mental State Examination. These observational findings do not establish causality and warrant further investigation.

## 1. Introduction

Cognitive decline is a major and escalating public-health challenge in aging societies, driving disability and substantial direct and indirect costs to health systems, families, and economies.^[1,2]^ It frequently coexists with other geriatric syndromes, amplifying functional loss and care dependence.^[3,4]^ The progressive neurodegenerative nature of Alzheimer disease underscores the imperative for timely prevention and early intervention.^[2,5]^ Notably, Chinese surveillance reports a post-COVID rise in cognitive impairment from 4.3% to 6.8%, highlighting older adults’ vulnerability to external shocks.^[1]^

Emerging work positions oral health, particularly tooth loss, as a potentially modifiable correlate of cognitive aging. Prior cohort studies have linked fewer remaining teeth or edentulism with dementia or poorer cognitive trajectories,^[6,7]^ while nutritional studies suggest that tooth loss and chewing difficulty may restrict food choices and reduce dietary diversity.^[8–11]^ However, whether dietary diversity helps explain the tooth–cognition association in a longitudinal Chinese cohort remains insufficiently characterized. We therefore hypothesized that a greater number of natural teeth would be associated with lower risk of incident cognitive impairment and that Dietary Diversity Score (DDS) would partially mediate this association.

## 2. Material and methods

### 2.1. Study population

Data were drawn from the Chinese Longitudinal Healthy Longevity Survey (CLHLS), a nationwide prospective cohort administered by Peking University. We used 2008 as baseline because this wave provided the required baseline tooth count, cognitive, dietary, and covariate information, and follow-up cognitive assessments were available in 2011, 2014, and 2018. The study period therefore allowed up to 10 years of follow-up for incident cognitive impairment.

Figure 1 shows the participant selection flowchart. From 16,954 CLHLS participants aged ≥65 years in 2008, we excluded participants with baseline cognitive impairment defined as Mini-Mental State Examination (MMSE) < 24 (n = 5251), missing baseline cognitive status (n = 3902), missing baseline number of natural teeth (n = 36), or no available cognitive assessment in all follow-up waves (n = 3401). The final analytic sample included 4364 participants with complete baseline exposure data and at least 1 follow-up cognitive assessment.

**Figure 1.**
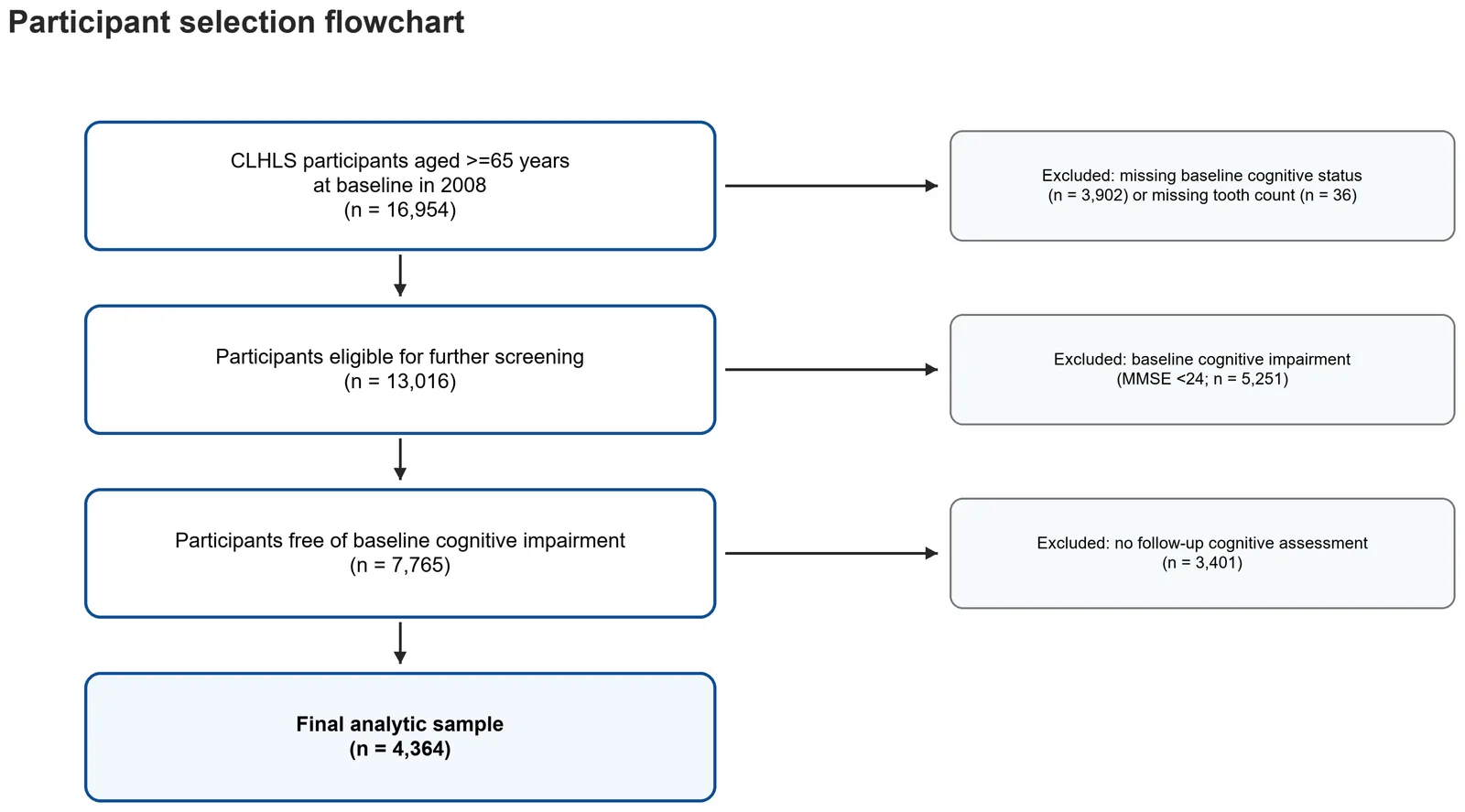
Flowchart of participant selection. CLHLS = Chinese Longitudinal Healthy Longevity Survey, MMSE = Mini-Mental State Examination.

### 2.2. Assessment of number of natural teeth

The number of natural teeth was ascertained during a structured, in-home assessment by trained investigators. Participants self-reported their count using the standardized item, “How many natural teeth do you have? (excluding false teeth).” The variable therefore reflects self-reported natural tooth count only; denture use, functional dentition, and clinical periodontal status were not available in the current analytic dataset.

### 2.3. Assessment of incident cognitive decline

Global cognition was assessed with the culturally adapted Chinese MMSE (total score 0–30), covering orientation, registration, attention or calculation, recall, and language. The MMSE was administered at baseline and each follow-up by trained investigators using standardized procedures.^[12–14]^ Participants with MMSE <24 at baseline were excluded. Among those free of cognitive impairment at baseline, incident cognitive impairment was defined as the first follow-up assessment with MMSE <24.^[15]^

### 2.4. Assessment of mediating variable

Trained personnel conducted face-to-face interviews and assessed baseline DDS using a food frequency questionnaire covering 9 food categories: meat, fish and seafood, eggs, legumes, fruits, salted vegetables, tea, garlic, and fresh vegetables. Intake frequency for each category was recorded as “regularly or almost daily,” “occasionally,” or “rarely or never.” DDS was defined as the count of categories reported as “regularly or almost daily,” with a possible range of 0 to 9. Cereals and edible oils were excluded because they are consumed nearly daily by most Chinese adults.^[11,16]^ Because DDS and tooth count were both measured at baseline, mediation results were interpreted as exploratory.

### 2.5. Assessment of covariates

To reduce confounding, we adjusted for baseline sociodemographic characteristics, lifestyle behaviors, and chronic diseases available in the analytic dataset. Covariates included age, sex, years of education, marital status, smoking status, drinking status, hypertension, diabetes, and stroke. DDS was not included in the primary total-effect Cox model because it was evaluated as a potential mediator.

### 2.6. Statistical analysis

Continuous variables were summarized as means and standard deviations or medians and interquartile ranges, as appropriate; categorical variables were summarized as counts and percentages. Time-to-event was calculated from baseline in 2008 to the first wave with MMSE <24 or to the last available cognitive assessment. Cox proportional hazards models estimated hazard ratios (HRs) and 95% confidence intervals (CIs) for incident cognitive impairment. Tooth count was analyzed per interquartile range (IQR) increase (20 teeth) and by quartiles (Q1, 0–1; Q2, 2–9; Q3, 10–21; Q4, ≥22 teeth). Model 1 was unadjusted; model 2 adjusted for age and sex; model 3 further adjusted for education, marital status, smoking, drinking, hypertension, diabetes, and stroke. A separate sensitivity/direct-effect model additionally adjusted for DDS. The proportional hazards assumption was assessed using Schoenfeld residuals.

Restricted cubic splines were used to evaluate the shape of the association between tooth count and incident cognitive impairment, with knots placed at 0, 5, 18, and 30 teeth and 10 teeth as the reference value. Overall and nonlinear associations were evaluated using likelihood-ratio tests. Subgroup analyses were performed by sex, marital status, smoking, drinking, hypertension, diabetes, stroke, age (<70 vs ≥70 years), DDS (<3 vs ≥3), and education (<6 vs ≥6 years), with multiplicative interaction terms. Exploratory mediation analysis used mean follow-up MMSE as the continuous outcome, baseline DDS as the mediator, baseline tooth count as the exposure, and adjusted for baseline MMSE and model 3 covariates; 10,000 bootstrap samples were used to estimate CIs. All analyses were conducted using R version 4.5.1 (R Foundation for Statistical Computing), and a 2-sided *P* < .05 was considered statistically significant.

## 3. Result

### 3.1. Characteristics of the study population according to incident cognitive impairment

This longitudinal analysis included 4364 participants free of cognitive impairment at baseline, among whom 1835 (42.0%) developed incident cognitive impairment during follow-up. The median follow-up was 6 years, contributing 24,585 person-years. Baseline MMSE was 28.18 ± 1.76. Participants who developed incident cognitive impairment were older, had fewer years of education, lower DDS, and fewer natural teeth at baseline than those who did not develop incident cognitive impairment (Table 1).

**Table 1:** Baseline characteristics of the study participants grouped by incident cognitive impairment during follow-up.

Variables	Total (n = 4364)	No incident cognitive impairment (n = 2529)	Incident cognitive impairment (n = 1835)	*P*
Number of natural teeth	12.45 ± 10.66	13.50 ± 10.86	11.01 ± 10.21	<.001
Baseline MMSE score	28.18 ± 1.76	28.38 ± 1.70	27.92 ± 1.81	<.001
Age	78.20 ± 9.53	77.01 ± 9.28	79.85 ± 9.63	<.001
Years of education	3.28 ± 3.91	3.91 ± 4.12	2.41 ± 3.43	<.001
DDS	2.61 ± 1.61	2.71 ± 1.65	2.46 ± 1.55	<.001
Sex				<.001
Male	2370 (54.31)	1510 (59.71)	860 (46.87)	
Female	1994 (45.69)	1019 (40.29)	975 (53.13)	
Marital				<.001
Nonmarried	1870 (42.85)	985 (38.95)	885 (48.23)	
Married	2494 (57.15)	1544 (61.05)	950 (51.77)	
Smoke				<.001
Nonsmoker	2553 (58.50)	1384 (54.73)	1169 (63.71)	
Current smoker	1074 (24.61)	682 (26.97)	392 (21.36)	
Former smoker	737 (16.89)	463 (18.31)	274 (14.93)	
Drink				<.001
Nondrinker	2777 (63.63)	1565 (61.88)	1212 (66.05)	
Former drinker	616 (14.12)	346 (13.68)	270 (14.71)	
Current drinker	971 (22.25)	618 (24.44)	353 (19.24)	
Hypertension				.210
Yes	1377 (31.55)	779 (30.80)	598 (32.59)	
No	2987 (68.45)	1750 (69.20)	1237 (67.41)	
Diabetes				.157
Yes	249 (5.71)	155 (6.13)	94 (5.12)	
No	4115 (94.29)	2374 (93.87)	1741 (94.88)	
Stroke				.860
Yes	661 (15.15)	381 (15.07)	280 (15.26)	
No	3703 (84.85)	2148 (84.93)	1555 (84.74)	

*P*-values were calculated from chi-square tests (categorical variables), rank-sum tests (non-normally distributed continuous variables), or *t*-tests (normally distributed continuous variables).

DDS = Dietary Diversity Scores, MMSE = Mini-Mental State Examination.

Baseline MMSE scores and DDS increased across higher quartiles of natural tooth count (Table 2). Participants in the highest tooth-count quartile (Q4, ≥22 teeth) were younger and had more years of education than those in lower quartiles, indicating the need for careful multivariable adjustment.

**Table 2:** Baseline characteristics of the study participants grouped by quartiles of the number of natural teeth.

Variables	Q1: 0–1 (n = 1029)	Q2: 2–9 (n = 993)	Q3: 10–21 (n = 1213)	Q4: ≥22 (n = 1129)	*P*
Baseline MMSE score	27.90 ± 1.82	27.90 ± 1.83	28.23 ± 1.76	28.65 ± 1.54	<.001
Age	82.86 ± 9.61	81.52 ± 9.03	76.64 ± 8.55	72.73 ± 7.41	<.001
Years of education	2.64 ± 3.54	2.63 ± 3.69	3.20 ± 3.97	4.51 ± 4.08	<.001
DDS	2.46 ± 1.51	2.45 ± 1.64	2.58 ± 1.56	2.91 ± 1.69	<.001
Sex					<.001
Male	490 (47.62)	536 (53.98)	637 (52.51)	707 (62.62)	
Female	539 (52.38)	457 (46.02)	576 (47.49)	422 (37.38)	
Marital					<.001
Nonmarried	554 (53.84)	540 (54.38)	494 (40.73)	282 (24.98)	
Married	475 (46.16)	453 (45.62)	719 (59.27)	847 (75.02)	
Smoke					<.001
Nonsmoker	613 (59.57)	598 (60.22)	741 (61.09)	601 (53.23)	
Current smoker	248 (24.10)	224 (22.56)	272 (22.42)	330 (29.23)	
Former smoker	168 (16.33)	171 (17.22)	200 (16.49)	198 (17.54)	
Drink					<.001
Nondrinker	713 (69.29)	637 (64.15)	765 (63.07)	662 (58.64)	
Former drinker	122 (11.86)	160 (16.11)	167 (13.77)	167 (14.79)	
Current drinker	194 (18.85)	196 (19.74)	281 (23.17)	300 (26.57)	
Hypertension					<.001
Yes	292 (28.38)	290 (29.20)	400 (32.98)	395 (34.99)	
No	737 (71.62)	703 (70.80)	813 (67.02)	734 (65.01)	
Diabetes					<.001
Yes	52 (5.05)	44 (4.43)	64 (5.28)	89 (7.88)	
No	977 (94.95)	949 (95.57)	1149 (94.72)	1040 (92.12)	
Stroke					.28
Yes	152 (14.77)	134 (13.49)	190 (15.66)	185 (16.39)	
No	877 (85.23)	859 (86.51)	1023 (84.34)	944 (83.61)	

*P* values were calculated from chi-square tests (categorical variables) or rank-sum tests (non-normally distributed continuous variables).

DDS = Dietary Diversity Scores, MMSE = Mini-Mental State Examination.

### 3.2. Association between number of natural teeth and incident cognitive impairment

Table 3 shows the Cox proportional hazards regression results for the association between the baseline number of natural teeth and incident cognitive impairment. The proportional hazards assumption was not violated for tooth count based on Schoenfeld residuals (*P* = .233). In the crude model, each IQR increase in tooth count was associated with a lower risk of incident cognitive impairment (HR = 0.63; 95% CI: 0.57–0.68; *P* < .001). The association remained after adjustment for age and sex (HR = 0.84; 95% CI: 0.76–0.92; *P* < .001) and in the fully adjusted model 3 (HR = 0.86; 95% CI: 0.78–0.95; *P* = .002). Compared with Q1 (0–1 teeth), Q4 (≥22 teeth) was associated with a lower risk in model 3 (HR = 0.81; 95% CI: 0.70–0.93; *P* = .004). A sensitivity/direct-effect model further adjusting for DDS yielded a similar association per IQR increase (HR = 0.87; 95% CI: 0.79–0.96; *P* = .005). Restricted cubic spline analysis indicated an overall association (*P* = .013) without evidence of nonlinearity (*P* = .467) (Fig. 2).

**Table 3:** Association between the number of natural teeth and incident cognitive impairment. Results are presented as HR (95% CI).

Variables	Model 1	*P*	Model 2	*P*	Model 3	*P*
Number of teeth (per IQR, 20 teeth)	0.63 (0.57–0.68)	<.001	0.84 (0.76–0.92)	<.001	0.86 (0.78–0.95)	.002
Number of teeth Quantile						
Q1	Reference		Reference		Reference	
Q2	0.93 (0.82–1.06)	.265	0.98 (0.87−1.12)	.802	0.96 (0.85−1.10)	.573
Q3	0.74 (0.65–0.84)	<.001	0.93 (0.82–1.06)	.259	0.92 (0.81–1.05)	.207
Q4	0.53 (0.46–0.61)	<.001	0.78 (0.68–0.91)	.001	0.81 (0.70–0.93)	.004

Model 1 was the crude model; model 2 was adjusted for age and sex; model 3 was further adjusted for years of education, marital status, smoking status, drinking status, hypertension, diabetes, and stroke. DDS was not included in model 3 because it was evaluated as a potential mediator; a sensitivity/direct-effect model further adjusting for DDS yielded HR = 0.87; 95% CI: 0.79–0.96; *P* = .005 per IQR increase. In this dataset, 1 IQR increase corresponded to 20 natural teeth.

CI = confidence interval, DDS = Dietary Diversity Score, HR = hazard ratio, IQR = interquartile range.

**Figure 2.**
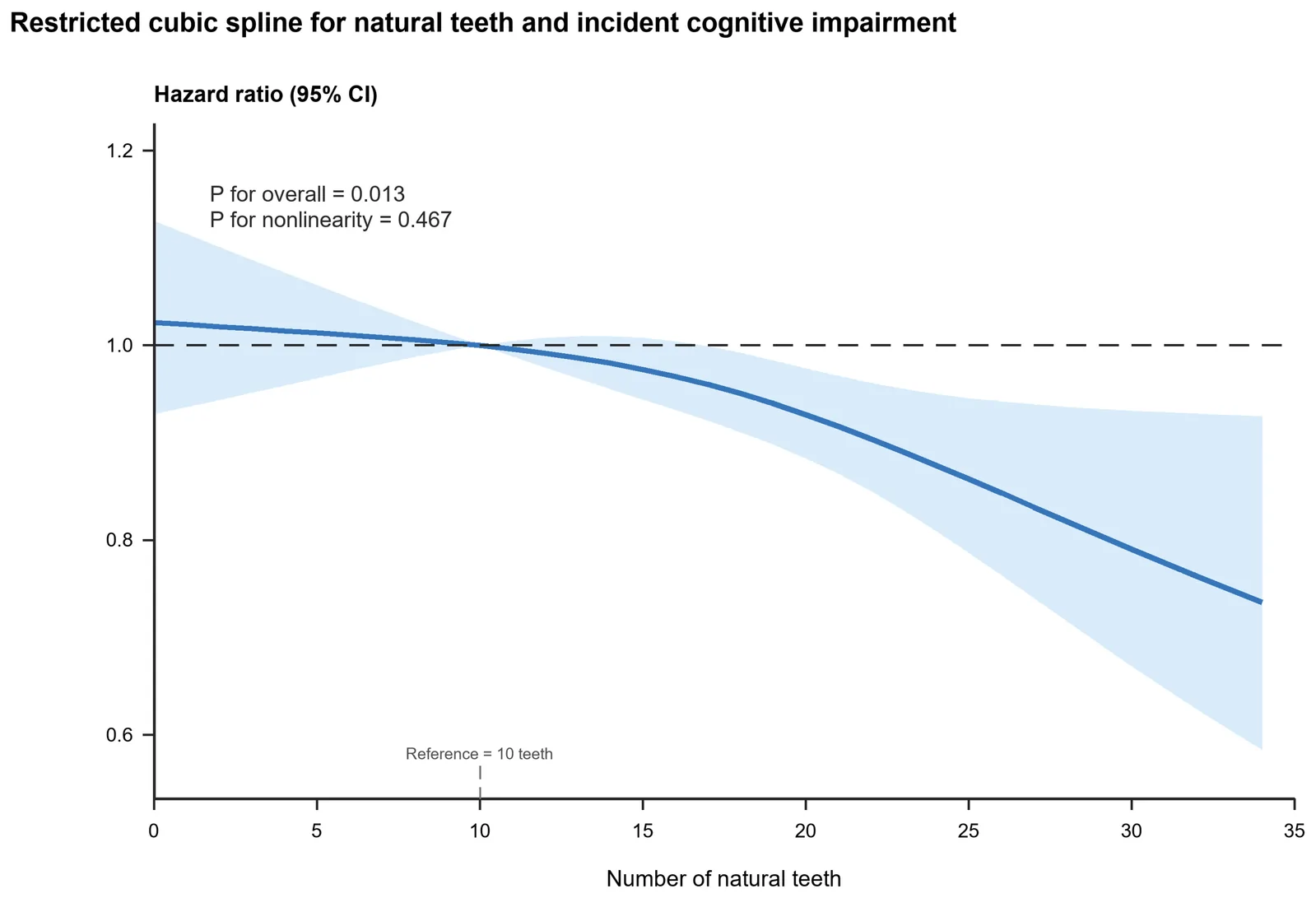
Restricted cubic spline of the association between the number of natural teeth and incident cognitive impairment. The model was adjusted for age, sex, years of education, marital status, smoking status, drinking status, hypertension, diabetes, and stroke; knots were placed at 0, 5, 18, and 30 teeth, with 10 teeth as the reference. CI = confidence interval.

### 3.3. Stratified analysis

Figure 3 shows the updated stratified analyses. The inverse association between tooth count and incident cognitive impairment was generally consistent across subgroups, although several subgroup estimates were imprecise. Age showed evidence of effect modification (*P* for interaction = .043). Specifically, each IQR increase in tooth count was associated with a lower risk among participants aged ≥70 years (HR = 0.73; 95% CI: 0.66–0.82; *P* < .001), whereas the association was not statistically significant among participants aged <70 years (HR = 0.95; 95% CI: 0.75–1.19; *P* = .642).

**Figure 3.**
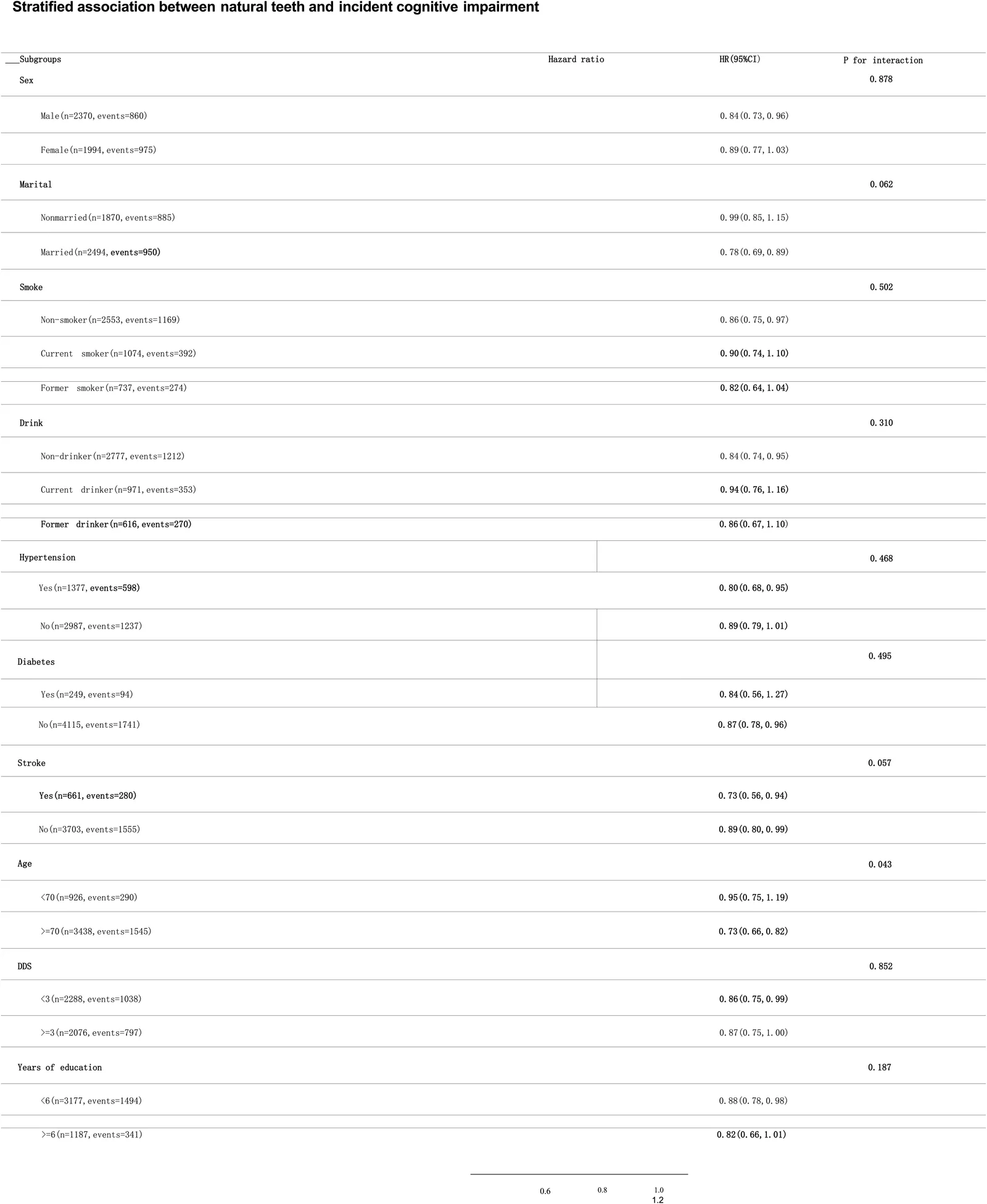
Forest plot of stratified analyses for the association between the number of natural teeth and incident cognitive impairment. CI = confidence interval, DDS = Dietary Diversity Score, HR = hazard ratio.

### 3.4. Mediation analyses

Table 4 presents the exploratory mediation analysis in which mean follow-up MMSE was treated as a continuous outcome. After adjustment for baseline MMSE and the covariates included in model 3, a higher number of natural teeth was positively associated with mean follow-up MMSE, although the magnitude of this total association was modest (total effect = 0.0119; 95% CI: 0.0011–0.0226; *P* = .048). When DDS was included as a potential mediator, the direct effect of tooth count on mean follow-up MMSE was attenuated and did not reach conventional statistical significance (direct effect = 0.0107; 95% CI: −0.0001 to 0.0215; *P* = .076). The indirect effect through DDS was small but statistically detectable (indirect effect = 0.0012; 95% bootstrap CI: 0.0001–0.0024; *P* = .038), suggesting that dietary diversity may explain a limited part of the association between natural tooth retention and subsequent cognitive performance. Mediation path coefficients are presented in Figure 4.

**Table 4:** Exploratory mediation of the association between the number of natural teeth and mean follow-up MMSE through DDS.

Path	Effect	Estimate	*P*	Boot 95% CI	Interpretation
Number of natural teeth-DDS-mean follow-up MMSE	Indirect (ab)	0.0012	.038	0.0001 to 0.0024]	Small exploratory indirect effect
Number of natural teeth-DDS-mean follow-up MMSE	Direct (c’)	0.0107	.076	−0.0001 to 0.0215	Not statistically significant at *P* < .05
Number of natural teeth-DDS-mean follow-up MMSE	Total (c)	0.0119	.048	0.0011 to 0.0226	Small total association with mean follow-up MMSE
Number of natural teeth-DDS-mean follow-up MMSE	Proportion mediated	9.92%	—	−1.45% to 53.19%	Wide bootstrap interval; interpret cautiously

Exploratory linear mediation models adjusted for baseline MMSE and model 3 covariates. Number of bootstrap samples for bias-corrected bootstrap confidence intervals: 10,000. Because tooth count and DDS were both measured at baseline, temporal ordering should be interpreted cautiously.

CI = confidence interval, DDS = Dietary Diversity Score, MMSE = Mini-Mental State Examination.

**Figure 4.**
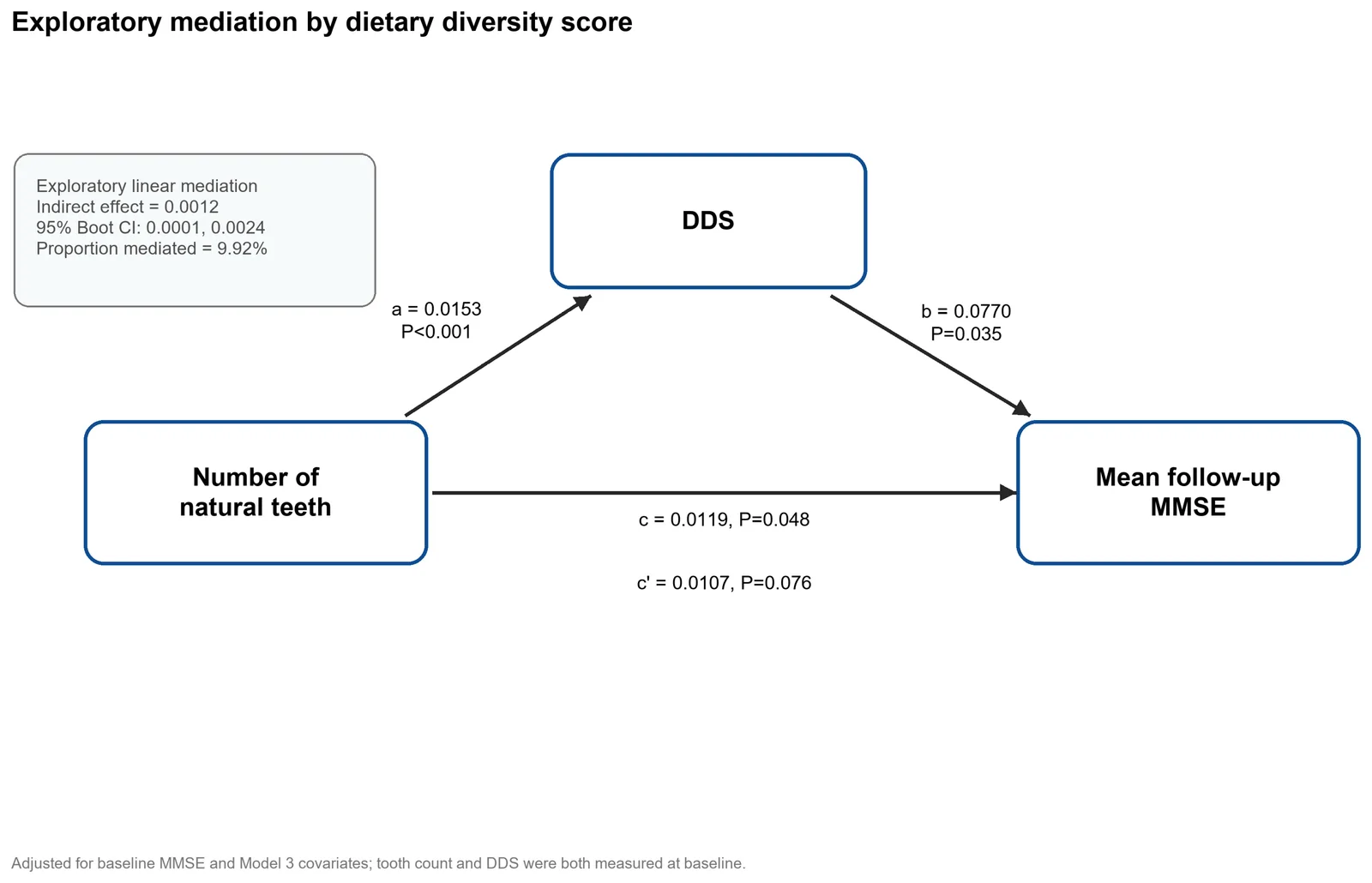
Path diagram of the exploratory mediating effect of DDS on the association between the number of natural teeth and mean follow-up MMSE. CI = confidence interval, DDS = Dietary Diversity Score, MMSE = Mini-Mental State Examination.

### 3.5. Sensitivity analyses

Sensitivity analyses yielded generally consistent findings (Table 5). After additional adjustment for DDS, each IQR increase in the number of natural teeth remained associated with a lower risk of incident cognitive impairment (HR = 0.87; 95% CI: 0.79–0.96; *P* = .005). When tooth count was categorized into quartiles, participants in the highest quartile had a significantly lower risk of incident cognitive impairment compared with those in the lowest quartile (HR = 0.81; 95% CI: 0.70–0.93; *P* = .004).

**Table 5:** Sensitivity analyses for the association between the number of natural teeth and incident cognitive impairment.

Analysis	Variables	Estimate	*P*
Main analysis	Number of teeth, per IQR increase (20 teeth)	0.86 (0.78−0.95)	.002
Additional adjustment for DDS	Number of teeth, per IQR increase (20 teeth)	0.87 (0.79−0.96)	.005
Categorical analysis	Q1: 0–1 teeth	Reference	
	Q2: 2–9 teeth	0.96 (0.85−1.10)	.573
	Q3: 10–21 teeth	0.92 (0.81−1.05)	.207
	Q4: ≥22 teeth	0.81 (0.70−0.93)	.004

DDS = Dietary Diversity Score, IQR = interquartile range, Q1–Q4 = quartile 1–quartile 4.

## 4. Discussion

In this longitudinal study of older Chinese adults, greater retention of natural teeth was associated with a lower risk of incident cognitive impairment during follow-up. This association remained evident after adjustment for age, sex, years of education, marital status, smoking status, drinking status, hypertension, diabetes, and stroke, and was similar after additional adjustment for dietary diversity. In the fully adjusted model, each interquartile range increase in the number of natural teeth was associated with a 14% lower risk of incident cognitive impairment (HR = 0.86; 95% CI: 0.78–0.95). Restricted cubic spline analysis supported an overall inverse association without strong evidence of nonlinearity, suggesting a broadly monotonic pattern across the observed range of tooth count. Subgroup analyses suggested a possible stronger association among participants aged 70 years or older, whereas most other interaction tests were not statistically significant. Exploratory mediation analysis indicated that dietary diversity may account for only a small estimated proportion of the association.

The observed inverse relationship between tooth number and incident cognitive impairment is consistent with prior studies linking oral health with dementia and cognitive aging.^[17,18]^ Several pathways may be biologically plausible, including impaired mastication,^[19–22]^ altered dietary intake, systemic inflammation, vascular dysfunction,^[23,24]^ and reduced sensory stimulation.^[25,26]^ However, the present epidemiological data cannot verify specific molecular mechanisms such as microglial pyroptosis or Cyclic GMP-AMP Synthase/Stimulator of Interferon Genes signaling^[27]^; these mechanisms should be viewed as hypotheses from prior experimental literature rather than mechanisms demonstrated by this study.

The exploratory mediation results support the possibility that dietary diversity contributes modestly to the oral health–cognition association. Tooth loss may constrain food choices and reduce intake of diverse foods, which in turn may influence cognitive health through nutritional, vascular, inflammatory, or metabolic pathways. Nevertheless, the estimated indirect effect was small, DDS was measured at the same baseline wave as tooth count, and the primary endpoint was incident cognitive impairment rather than continuous MMSE. Therefore, the mediation finding should be presented as exploratory and hypothesis-generating.

The age-stratified results require a more cautious interpretation than originally stated. The updated analysis suggested that the association was evident among participants aged ≥70 years, whereas the <70-year subgroup did not show a statistically significant association. Although the interaction test reached statistical significance (*P* = .043), subgroup analyses were exploratory and may be sensitive to sample size, event distribution, and multiple testing. These findings should therefore be interpreted as evidence of possible age heterogeneity rather than proof that tooth retention is uniquely important in 1 age group.

Several limitations should be considered. First, the observational design precludes causal inference, and reverse causality is possible: individuals in prodromal stages of cognitive impairment may have poorer oral hygiene, reduced dental care, or dietary changes before diagnosis. Second, tooth count and DDS were self-reported, and no clinical dental examination, periodontal status, denture use, chewing ability, portion size, or nutrient-density information was available in the current analytic dataset. Third, although we adjusted for available sociodemographic characteristics, lifestyle behaviors, and chronic diseases, residual confounding by income and other socioeconomic factors not included in the models cannot be excluded. Fourth, competing risk of death could not be assessed because death status and cause-specific censoring information were not included in the current dataset. Fifth, the dataset contained only the final analytic sample, so we could not compare baseline characteristics between included and excluded participants; selection bias and survivor bias may therefore persist. Finally, cognitive impairment was defined using a fixed MMSE <24 cutoff rather than education-specific cutoffs or repeated-measures cognitive trajectories, which may have introduced outcome misclassification.

## 5. Conclusion

In conclusion, greater natural tooth retention was associated with a lower risk of incident cognitive impairment among older Chinese adults in the CLHLS. Dietary diversity may account for a small part of this association, but the mediation finding is exploratory. These results support further longitudinal and interventional studies with clinical dental examinations, denture and mastication assessments, richer socioeconomic and behavioral covariates, and competing-risk methods before drawing causal or policy conclusions.

## Author contributions

**Conceptualization:** Qihang Chen, Xiaobao Dang.

**Writing – original draft:** Qihang Chen, Xiaobao Dang, Jingke Liu, Zhen Zhu.

**Writing – review & editing:** Qihang Chen.

**Formal analysis:** Xiaobao Dang.

**Funding acquisition:** Xiaobao Dang.

**Investigation:** Jingke Liu.

**Methodology:** Jingke Liu, Zhen Zhu.

**Project administration:** Jingke Liu.

**Resources:** Zhen Zhu.
